# Factors associated with enrollment for community-based health insurance scheme in Western Ethiopia: Case-control study

**DOI:** 10.1371/journal.pone.0252303

**Published:** 2021-06-10

**Authors:** Meseret Belete Fite, Kedir Teji Roba, Bedasa Taye Merga, Belay Negash Tefera, Gemechu Ayela Beha, Temesgen Tafesse Gurmessa

**Affiliations:** 1 Department of Public Health, Institute of Health Science, Wollega University, Nekemte, Ethiopia; 2 School of Public Health, College of Medicine and Health Sciences, Haramaya University, Harar, Ethiopia; 3 Maternal and Child Health Services Team, Gida Ayana District Health Office, Ayana, Ethiopia; 4 Malaria and NTD Directorate, Armauer Hansen Research Institute, Ministry of Health, Addis Ababa, Ethiopia; Health Services Academy Islamabad Pakistan, PAKISTAN

## Abstract

**Introduction:**

Modern health services utilization in developing countries has continued low. Financial shortage to access health-care services might be averted by stirring from out-of-pocket payment for health care at the time of use. The government of Ethiopia; depend greatly on foreign aid (50%) and out-of-pocket payments (34%) to fund health services for its population. This study was aimed to identify factors associated with households’ enrollment to CBHI scheme membership.

**Methods:**

Case-control study design was conducted from May 18–July 27, 2019 among 332 participants (166 enrolled and 166 non-enrolled to CBHI scheme). Simple random sampling technique was used to select the study participants. Bi-variable and multivariable logistic regression model were fitted to identify factors associated with enrollment to community based health insurance. Adjusted odds ratio (AOR) with 95% CI was used to report association and significance was declared at P<0.05.

**Result:**

A total of 332 (100% response rate) were involved in the study. Educational status (College and above, AOR = 3.90, 95%CI; 1.19, 12.75), good awareness about CBHI scheme (AOR = 21.595, 95% CI; 7.561, 61.681), affordability of premium payment (AOR = 3.403, 95% CI; 5.638–4.152), wealth index {(Poor, AOR = 2.59, 95%CI; 1.08, 6.20), (Middle, AOR = 4.13, 95%CI; 1.11, 15.32)} perceived health status (AOR = 5.536; 95% CI; 1.403–21.845), perceived quality of care (AOR: 21.014 95%CI; 4.178, 105.686) and treatment choice (AOR = 2.94, 95%CI; 1.47, 5.87) were factors significantly associated with enrollment to CBHI.

**Conclusion:**

Enrolment to CBHI schemes is influenced by educational level, awareness level, affordability of premium, wealth index, perceived health status, perceived quality of care and treatment choice. Implementation strategies aimed at raising community awareness, setting affordable premium, and providing quality healthcare would help in increasing enrollment of all eligible community groups to the CBHI scheme.

## Introduction

The 2030 Sustainable Development Goals (SDG) emphasizes ensuring all peoples’ access to quality healthcare services without financial hardship [1]. Modern health services utilization in developing countries has continued to be low. These countries have insufficient access to and utilization of modern health care services due to shortage of finance [2]. Worldwide, yearly estimation shows that 44 million household’s face disastrous healthcare expenses, whereas 25 million households are unplanned because of direct health care payments [3]. Financial shortage to access health-care services might be averted by moving from out-of-pocket payment (OOP). Community-Based Health Insurance (CBHI) is a platform accomplished and functioned by a community-based organization, exclusive of government or a private for-profit company, that offers risk-pooling to cover the expenses of health care services [4].

A number of counties have contained Community-Based health insurance as part of general health financing strategy [5].Underprivileged people are excluded from formal insurance system in most of developing countries. Hence, recently a plenty of assessment have made suggestion on possible of community health insurance in escalation access to care and deal financial shield alongside the expenses of illness for these Underprivileged people [6].

Ethiopia is the second largest county, next to Nigeria in terms of population size in Africa. However the country ranks low in access to modern healthcare services compared to African countries [7]. Even though Ethiopia has been implementing the primary health-care approach since mid-1970s, the country was quietly continued with a basic challenge comprised: insufficient coverage of services, disproportion of access, inadequate quality of care, and high out-of-pocket expenditure [8].

In Ethiopia, CBHI scheme have been introduced to reduce the financial shock due to unexpected and irregular out-of-pocket payments and to increase the resource pooling for healthcare that would improve access and utilizations of health services [9,10]. The government of Ethiopia depend greatly on outdoor supporters (50%) and out-of-pocket payments (34%) to fund health services for its population [10,11].

Given the significant importance of community based health insurance in improving access to and utilization of healthcare service, in 2016 only 5% of Ethiopian were enrolled for CBHI schemes membership [12]. Having information on the determinants of enrollment into the CBHI scheme would help in devising strategies that promote communities’ enrollment into the CBHI scheme. Hence, this study aimed at assessing determinants of enrollment into CBHI scheme membership.

## Methods

### Study design, period and study setting

Case-control study was conducted from May 18–July 27, 2019 in Gida Ayana district. Gida Ayana is one of the districts in east Wollega Zone, Oromia regional state, Western Ethiopia. Ayana town is the administrative town of the District which is located 110 km from Nekemte (the Zonal capital), 443 km from Addis Ababa. Administratively, the district is divided into 7 urban Kebeles and 24 rural kebeles. The district has 145,712 populations of which 106,038(72.772%) were living in rural areas and directly engaged in agriculture. There are 3 government health centers, 23 governmental health post and six private health facilities in the district. According to the District CBHI scheme office there were an estimated 30,357 households in the District among this, 14670(48.3%) households were enrolled in CBHI [13].

### Source population and study population

Households who were resided for at least six month in Gida Ayana district during the study period were source population and selected households from 6 kebeles were study population. Household heads employed in formal sector and or covered by other insurance scheme for health and unable to communicate (Critically ill) during data collection were excluded from the study

### Sample size determination, *sampling procedure and technique*

The sample size was estimated by using Epi info, version 7.0. Similar study conducted in Achefer District, North- West, Ethiopia: P1 = 35%, P2 = 20% CBHI non-member and member households respectively [14], with the following assumptions: 80% statistical power with a level of significance at 95%, insured to uninsured ratio of 1:1 and 10% non-response rate was used. Accordingly, total a total sample size of 332 household s was calculated (166 members and 166 non-members).

The study participants were drawn using a multi-stage sampling technique. In the first stage, six Kebeles were selected randomly (lottery method) out of thirty one kebeles considering the representativeness of the kebeles. The second stage involved the selection of households from six kebeles. List of households for cases (enrolled) and controls (non-enrolled) obtained from each kebele’s administration household record list which was used as a sampling frame. The sample size was proportionally allocated for selected kebeles based on each kebele’s number of households. Then simple random sampling (lottery method) was employed to select the cases and controls by taking their list as a frame and labeling continuous numbering.

### Data collection tools and techniques

Data were collected using a structured interviewer administered questionnaire which is adapted from Ethiopian Demographic and Health Survey [12]. The questionnaire contains: Socio-demographic characteristics, health care access-related factors and Health perception and health-care needs variables. The questionnaire was prepared first in English and it was translated in to local language (*Afaan Oromo*) and back to English to check for its consistency. Four BSc nurses and 2 Public Health officers were involved for data collection and supervision respectively. The supervisors were recruited to check daily for the completeness, clarity and consistency of the questionnaire and to give appropriate support during the data collection process.

### Data quality assurance

Two day training was given for each data collectors and supervisors. The training was also emphasized on the importance of the privacy and confidentiality of respondents. Regular supervision was given during data collection. Collected data was checked by supervisors before sent to the data entree on daily basis. Data collection material was pretested in 10% using similar set up at Kiramu district. To ensure the quality of data, collected data were evaluated daily for completeness and consistency.

### Data processing and analysis

Data were cleaned and edited after entering into Epi data version 5.3.1 and then exported to SPSS version 20 for analysis. Descriptive statistics were computed and presented using frequencies, proportions, summary statistics, graphs and tables. Variables that have P-value < 0.25 on bivariate analyses were entered in the multivariate logistic regression model to identify independent predictors of enrollment into CBHI. P<0.05 was considered as statistically significant. The strength of association and precision were examined using adjusted odds ratio at 95% confidence interval. The model fitness was checked by Hosmer and Lemeshow goodness of fit test.

### Ethical consideration

Ethical approval was secured from Santé Medical College (SMC), department of public health. A formal letter written from the university was submitted to CBHI Gida Ayana District health offices and the District administration offices to obtain their co-operation. Then support letter was written to each selected Kebeles. The purpose of the study was described to the study participants. All study participants were also informed about their right of not participating in the study at any time and written informed consent was obtained from the study participants. Privacy and confidentiality of collected information was assured.

## Result

### Socio-demographic characteristics

A total of 332 Households’ heads were looked over in this study; and the study response rate was 100%. Household’s socio-demographic characteristic by current CBHI scheme is described in (**Table 1**). Majority of households’ heads were male both in CBHI member s130 (78.3%) and non- members134 (80.7%). Regarding the marital status of the respondents most of households head were married in both CBHI member 154 (92.8%) and non-members 157(94.5%). More than half of households have 4–6 f family size both in CBHI members 104(62.7%) and non- member 86(51.8%). Greater proportion of sample households were protest region follower both in CBHI member 52(31.37%) and non-member 47(28.3%). Moreover, concerning occupational status, more than half of household’s head who are farmers; comprising 52(31.3%) and 113(68.1%) both in CBHI scheme members and non- members respectively.

**Table 1:** Socio-demographic characteristics and CBHI scheme membership of study participants in Gida Ayana district, East Wollega Zone, Western Oromia, Ethiopia, and May 2019.

Explanatory Variables	CBHI membership		Chi-squared, p-value
	Enrolled	Not enrolled	
**Sex of households’ heads**			
Female	36(10.8)	32(9.6)	X^2^ = 0.2959, P = 0.586
Male	130(39.2)	134(40.4)	
**Age**			
18–35	130(39.2)	112(33.7))	X^2^ = 8.7983, P = 0.012
36–44	35(10.5)	30(9.0)	
45–65	1(0.3)	24(7.2)	
**Income**			
Rich	17(5.1)	17(5.1)	X^2^ = 13.2083, P = 0.001
Middle	132(39.8)	107(32.2)	
Poor	17(5.1)	42(12.6)	
**Family Size**			
1–3	34(10.2)	29(8.7)	X^2^ = 8.7983, P = 0.012
4–6	104(31.3)	86(25.9)	
> 6	28(8.4)	51(15.4)	
**Religion**			
Orthodox	34(10.2)	46(13.9)	X^2^ = 2.9789, P = 0.395
Protestant	52(15.7)	47(14.2)	
Muslims	39(11.7)	31(9.3)	
Waqefata	41(12.3)	42(12.6)	
**Marital status**			
Married	154(46.4)	157(47.3)	X^2^ = 0.8471, P = 0.655
Widowed	5(1.5)	5(1.5)	
Divorced	7(2.1)	4(1.2)	
**Education**			
No education	62(18.8)	97(29.2)	X^2^ = 22.391, P = 0.000
Primary (1–8)	46(13.9)	31(9.3)	
Secondary (9–12)	31(9.3)	31(9.3)	
College & above	27(8.1)	7(2.1)	
**Occupation**			
Farmer	135(40.7)	113(34)	X^2^ = 17.028, P = 0.001
Employee	15(4.5)	11(3.3)	
Daily labor	4(2.1)	22(6.6)	
Merchant	12(3.6)	20(6)	

### Factors affecting enrolment for CBHI schemes membership

In the Bivariable analysis variables that were found be significant at p-value *<*0.25 with 95% CI selected were fitted for further analysis in multivariable logistics regression model to control confounders and to ascertain the effect of each independent variables on the likelihood of CBHI enrolment. A final multivariable logistic regression analysis was performed to assess the impact of a number of factors on the likelihood that household heads to enroll in CBHI.

Multivariable binary logistic regression model indicate respondents who had college and above education level were 3.90 times more likely to be enrolled into CBHI scheme compared to those with no education (AOR = 3.90, 95%CI; 1.19, 12.75).

Respondents with good awareness level on CBHI scheme membership were 4.74 times more likely to be enrolled for CBHI compared to those with poor awareness level (AOR = 4.74, 95% CI; 2.50, 8.99). Respondents who perceived premium of payment for CBHI as cheap were found to be 5.82 times more likely to be enrolled CBHI scheme membership compared to those who perceived premium of payment for CBHI as expensive (AOR = 5.82, 95% CI; 2.49, 13.60).

Households from poor and middle wealth index were more likely to be enrolled into CBHI compared to those from rich wealth index ((Poor, AOR = 2.59, 95%CI; 1.08, 6.20), (Middle, AOR = 4.13, 95%CI; 1.11, 15.32)).

Respondents who perceived their own health status as good during data collection were found to be 5.58 times more likely enrolled for CBHI scheme membership compared to those perceived as poor their own health status (AOR: 5.58, 95%CI: 2.87, 10.87).

Respondents who perceived high quality of care were found to be 8.37 times more likely enrolled in CBHI scheme membership compared to those perceived low quality of care (AOR = 8.37; 95% CI; 3.62, 19.38). Households whose treatment choice modern healthcare were 2.94 times more likely to be enrolled compared to those who treatment choice was traditional/home healing (AOR = 2.94, 95%CI; 1.47, 5.87) (**Table 2**).

**Table 2:** Multivariable analysis of binary logistic regression of independent factors of CBHI scheme enrolment of study participants in in Gida Ayana District, Oromia Region, Western Ethiopia 2019 (n = 332).

Variables	Not enrolled	Enrolled	COR (95% CI)	AOR (95% CI)
**Sex**				
Male	32	36	1	1
Female	134	130	0.86(0.50, 1.47)	0.52 (0.24, 1.15)
**Family size**				
1–3	29	34	1	1
4–6	86	104	1.03(0.58, 1.83)	1.38 (0.61, 3.11)
>6	51	28	0.47(0,24, 0.92)*	0.62 (0.18, 2.12)
**Educational status**				
No education	97	62	1	1
Primary(1–8)	31	46	2.32(1.33, 4.05)**	2.02 (0.94, 4.34)
Secondary(9–12)	31	31	1.56(0.87, 2.83)	1.61 (0.72, 3.62)
College & above	7	27	6.03(2.48, 14.70)**	3.90 (1.19, 12.75)*
**Awareness level**				
Poor awareness	95	38	1	1
Good awareness	71	128	4.50(2.80, 7.25)**	4.74 (2.50, 8.99)**
**Time to nearby health facility**				
less than 1hr	85	72	1	1
Above 1hr	81	94	1.37(0.89, 2.11)	1.27 (0.69, 2.33)
**Affordability of premium**				
Expensive	51	12	1	1
Moderate	25	14	2.38(0.96, 5.89)	2.28 (0.73, 7.13)
Cheap	90	140	6.61(3.34, 13.08)**	5.82 (2.49, 13.60)**
**Wealth index**				
Rich	42	17	1	1
Middle	107	132	2.05 (1.64, 5.65)**	2.59(1.08, 6.20)*
Poor	17	17	2.47 (1.03, 5.93)*	4.13(1.11, 15.32)*
**Perceived health status**				
Good	110	43	1	1
Poor	56	123	5.62 (3.50, 9.02)**	5.58 (2.87, 10.87)**
**Perceived healthcare quality**				
Poor	68	26	1	1
Medium	71	68	2.50(1.43, 4.39*	2.01 (0.97, 4.17)
Good	27	72	6.97(3.70, 13.12)**	8.37 (3.62, 19.38)**
**Treatment choice**				
traditional/home heal	58	32	1	1
Modern healthcare	108	134	2.25(1.36, 3.70)**	2.94 (1.47, 5.87)**
**History of illness in the past 6 months**				
Yes	85	85	1	1
No	81	81	1.00(0.65, 1.54)	1.19 (0.63, 2.23)
**Age**				
< = 35years	112	130	1	1
>35 years	54	36	0.57(0.35, 0.94)	0.63 (0.23, 1.74)

1 = reference

* = p<0.05

** = significant at p<0.001 in multivariable logistic regression.

## Discussion

This study identified factors affecting enrollment into CBHI scheme membership in Gida Ayana district. Educational status, awareness about CBHI, affordability of premium payment, wealth index, perceived health status, perceived quality of care, and treatment choice were independent determinants of CBHI enrollment.

Having a college and above level of education was positively associated with enrollment into CBHI schemes. This finding is in agreement with studies conducted in western Ethiopia and Senegal [15,16]. This could be because an educated people might understand the CBHI scheme principles, and benefit packages. The study revealed that respondents who had good awareness level on CBHI scheme were more likely to be enrolled into CBHI compared to those with poor awareness level. The finding is consistent with study conducted in Nigeria [17], Ethiopia [18]. This may be due to the fact that individuals with better information may ask details of the services and get more understandings of its advantage that drives them to be enrolled in CBHI. This implies that raising community awareness may help in the successful implementation of the scheme.

Affordability of premium for CBHI was found to be significantly associated with enrollment into CBHI schemes. This result is highly supported by evidence investigated in Ghana [19] and Mali [20]. Concurrently, finding of study conducted in North West Ethiopia [18], documented households who perceived amount of premium payment as affordable were more likely to enroll into CBHI. In Ethiopia [21], premium was collected from household for CBHI scheme membership at pre-defined fixed amount that is equal amount of money was charged to each household deprived of taking into account the capacity and features of the households including wealth or demographic factors. Hence, this might limit the enrollment of households for CBHI scheme membership.

The study revealed that households with poor and middle wealth index were more likely enroll into CBHI scheme compared to the rich wealth index. This result is supported by studies conducted in Ethiopia [22,23]. This might be explained by the target of CBHI scheme which mainly is increasing healthcare access of citizens recruited at informal sectors, and as a result the relative low premium payment [22].

Perceived quality of healthcare services is found to be factors significantly associated with enrollment into CBHI scheme. This finding is consistent with evidences from northwest Ethiopia and Nigeria [24,25]. This may be justified by, services with standard quality may attract more customers to utilize and enjoy the services. The same is true for CBHI scheme, the more the society perceive the quality of health services as good the more they will enroll into the scheme. Some studies report the same findings that support this study

Respondents who perceived their health status as poor had more probability of CBHI enrollment compared to those who claimed their health status as very good. And this may be justified as; household heads with perceived poor health status may believe on the importance of health service hence leading to health insurance enrollment. This finding is supported by other studies conducted in Bangladesh [26] and Mumbai [27].

Households whose treatment choice was modern healthcare had higher odds of enrollment into CBHI scheme as compared to those whose treatment choice was traditional or home healing. Similarly different studies revealed that those whose prefer modern healthcare as their first treatment choice utilize health service than those who prefer traditional healers [28]. Uninsured households refrain from facilities based healthcare (hospital, health center) and might increase attendance for traditional healer given lower service cost of healings and transportation compared to healthcare facilities did [29].

## Conclusion

The study identified factors associated with enrollment to CBHI scheme memberships. Enrollment to CBHI schemes is influenced by educational status, awareness level, affordability of premium, wealth status, perceived health status, perceived quality of care and treatment choice. Implementation strategies aimed at raising community awareness on the premium, its principle, and benefits packages of CBHI membership should be given a due emphasis to attract more eligible community groups. Affordability of the premium determines the decision to enroll and renewal of the membership, therefore, setting affordable premium would help in successful enrollment of all eligible community groups to the CBHI scheme. Provision of quality healthcare would also be a focus of program implementers to increase enrollment CBHI scheme. To have more insight on sociocultural determinants of enrollment into CBHI, the prospective researchers would consider both qualitative and quantitative research that involve broad geographic areas and with large sample size.

## Abbreviations

CBHI: Community-Based Health Insurance
EDHS: Ethiopian Demographic and Health Survey
WHO: World Health Organization
